# Electrophoretic Separation on an Origami Paper-Based Analytical Device Using a Portable Power Bank

**DOI:** 10.3390/s19071724

**Published:** 2019-04-10

**Authors:** Yu Matsuda, Katsunori Sakai, Hiroki Yamaguchi, Tomohide Niimi

**Affiliations:** 1 Department of Modern Mechanical Engineering, Waseda University, 3-4-1 Ookubo, Shinjuku-ku, Tokyo 169-8555, Japan; 2 Department of Micro-Nano Mechanical Science and Engineering, Nagoya University, Furo-cho, Chikusa, Nagoya, Aichi 464-8603, Japan; sakai.katsunori@d.mbox.nagoya-u.ac.jp (K.S.); hiroki@nagoya-u.jp (H.Y.)

**Keywords:** paper-based analytical device, electrophoresis, origami

## Abstract

The electrophoresis of ampholytes such as amino acids on a paper device is difficult because of the variation of pH distribution in time. On the basis of this observation, we propose a paper-based analytical device (PAD) with origami structure. By folding a filter paper, a low operation voltage of 5 V was achieved, where the power was supplied by a 5 V 1.5 A portable power bank through the USB type A receptacle. As a demonstration, we carried out the electrophoretic separation of pI markers (pI 5.5 and 8.7). The separation was achieved within 4 min before the pH distribution on the paper varied. Though the separation distance was small, it could be increased by expanding the origami structure. This result indicates that our proposed PAD is useful for electrophoretic separation on a paper device.

## 1. Introduction

Since the paper-based analytical device (PAD), which is also referred to as micro paper-based analytical device (μPAD), was first proposed by Whitesides and co-workers [1], many studies have been reported and reviewed [2,3,4,5,6,7,8,9,10,11]. As PAD has unique advantages, such as low-cost and being disposable and portable, it is a promising device for instant analyses. The analyses, which can be conducted on the paper, are limited compared with those possible on glass or resin (e.g., polydimethylsiloxane (PDMS) and polymethyl methacrylate (PMMA)) fluidic devices, because the fluid channel consists of a piece of paper. The disadvantages of PADs are its paper-thin channel structure and the difficulties in implementing a valve, a micro-relief structure, etc., in this channel structure. There are many reports that propose to enhance the function of PADs by implementing electrodes or an electric circuit. For example, electrochemical detection on PAD was achieved by screen-printed electrodes [12]. A stable reference electrode for electrochemical detection was also proposed [13]. The conductive pattern was implemented for thermo-fluidic control on PAD [14]. Separation on PAD is one of the key techniques for achieving more complex analysis. For example, amperometric detection was developed by combining chromatographic separation and gold electrodes on a polyester film [15] or paper [16]. Though this method is very simple and low-cost, it requires a long time. However, despite the above considerations, electrophoresis is a promising method of separation. There are many studies developing electrophoretic separation devices [17,18,19,20,21,22]. Electrophoretic separation on paper was carried out by applying the voltage of 800 V using an electrophoresis apparatus [17]. A paper strip, 200 mm long, on which the leading wires were placed, was prepared, and a sample solution was electrophoretically separated at a voltage of 100 V [18]. By stacking papers and manipulating the placement and dimensions of the conductors [23], which have several functions, such as channel, reservoir, and electrodes, a PAD for electrophoretic separation was introduced [19]. The applied separation voltage was 330 V in this PAD. A PAD consisting of paper and reservoirs (micropipette tips) was developed [20]. In this system, the electrodes were put into the reservoirs, and a voltage of 300 V was applied between the electrodes. These PADs are very useful for the separation of their target materials. However, the applied voltage necessary for the electrophoretic separation was high (>100 V); thus, a large and heavy power source equipment was required for the operation. This requirement limits the portability of PADs. Wu et al. [22] developed a PAD for simultaneous concentration and separation of proteins. In their PAD, the power for electrophoresis was supplied by a portable power bank and was boosted to 300 V by a DC–DC booster. Li et al. [24] proposed an origami PAD operating at a low voltage (10 V). In this study, inspired by this technique, we propose a PAD operating at an even lower voltage of 5 V with the power supplied by a portable power bank through a USB receptacle. The efficient electrophoretic separation of two materials can be conducted by migrating them in opposite directions; thus, we adopted a buffer solution whose pH value was between the pI values of the materials. This PAD can realize electrophoretic separation without loss of the portability which is one of the unique advantages of PAD.

## 2. Materials and Methods

**Figure 1:**
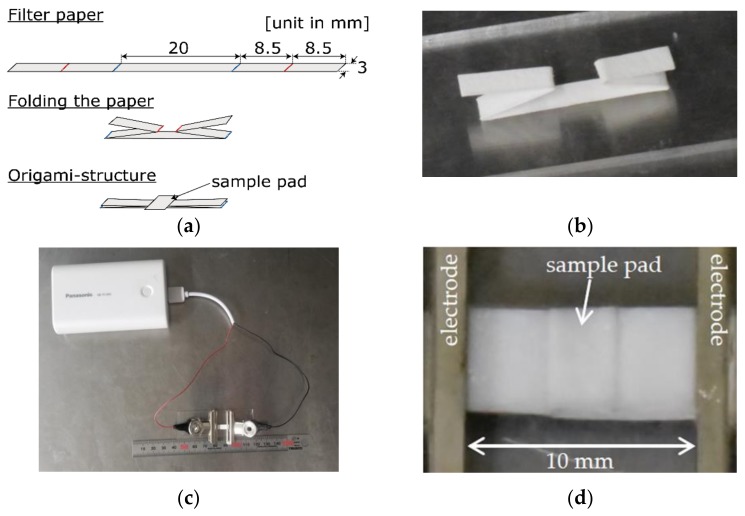
Proposed paper-based analytical device (PAD) device. (**a**) How to make the proposed origami structure. A sample pad was put on the center of the folded paper. (**b**) Photograph of the origami structure. (**c**) The origami PAD connected to a portable power bank. (**d**) Close-up photograph of the PAD. Bulldog clips were used as electrodes.

We prepared a piece of cellulose filter paper, 54 mm long and 3.0 mm wide (Whatman filter paper grade 3, GE Healthcare, Chicago, IL, USA) and folded it into an origami structure, as shown in Figure 1a,b. A piece of filter paper, 3.0 mm wide, on which we dropped a sample solution, was put on the center of the origami structure. Bulldog clips (very small, KURI-J16, Kokuyo, Osaka, Japan) were used as electrodes, as shown in Figure 1c,d. The distance between the electrodes was 10 mm. The power was supplied by a 5 V 1.5 A portable power bank (QE-PL302, Panasonic, Osaka, Japan) through a USB type A receptacle (Figure 1c). We also prepared a piece of pH test paper (pH range 1.0–14.0, GE Healthcare, Chicago, IL, USA) to visualize the pH distribution during the experiment; the voltage was applied by a DC power supply (PMM18-2.5DU, Kikusui Electronics, Yokohama, Japan) for long-time stable operation.

Sodium phosphate buffer solution (50 mM, pH 7.0) was prepared by mixing NaH_2_PO_4_ and Na_2_HPO_4_ stock solutions. Disodium hydrogen phosphate dehydrate (NaH_2_PO_4_·2H_2_O) and disodium hydrogen phosphate 12-Water (Na_2_HPO_4_·12H_2_O) were purchased from FUJIFILM Wako Pure Chemical Corporation (Osaka, Japan) and used to prepare the NaH_2_PO_4_ and Na_2_HPO_4_ solutions. The pH of the solution was measured by a pH meter (AS600, Asone, Osaka, Japan; measuring pH range 0.00 to 14.00 ± 0.01 pH).

As a sample solution, we prepared a mixture solution of pI markers (fluorescent IEF markers pI 5.5 and 8.7, Sigma-Aldrich, St. Louis, Missouri, USA). Both pI marker solutions were diluted to 0.3 mg/mL (diluted 10 times) with the buffer solution. The pI markers were illuminated by a UV lamp (wavelength: 365 nm, LUV-4, AS ONE, Osaka, Japan), and the resultant fluorescence was taken by a CMOS camera (Nikon 1S2, Tokyo, Japan).

## 3. Results and Discussion

### 3.1. pH Distribution

**Figure 2:**
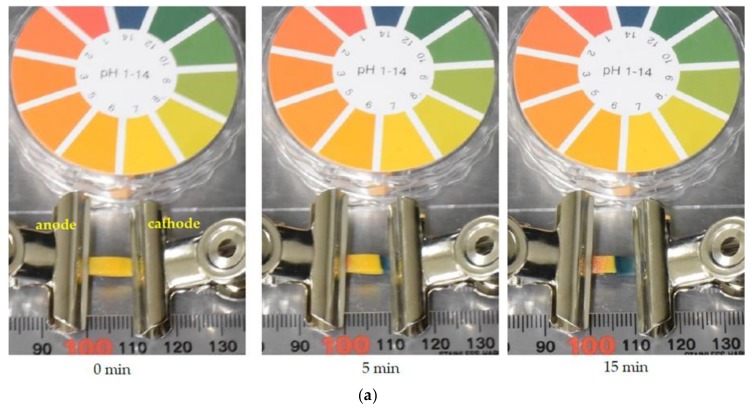

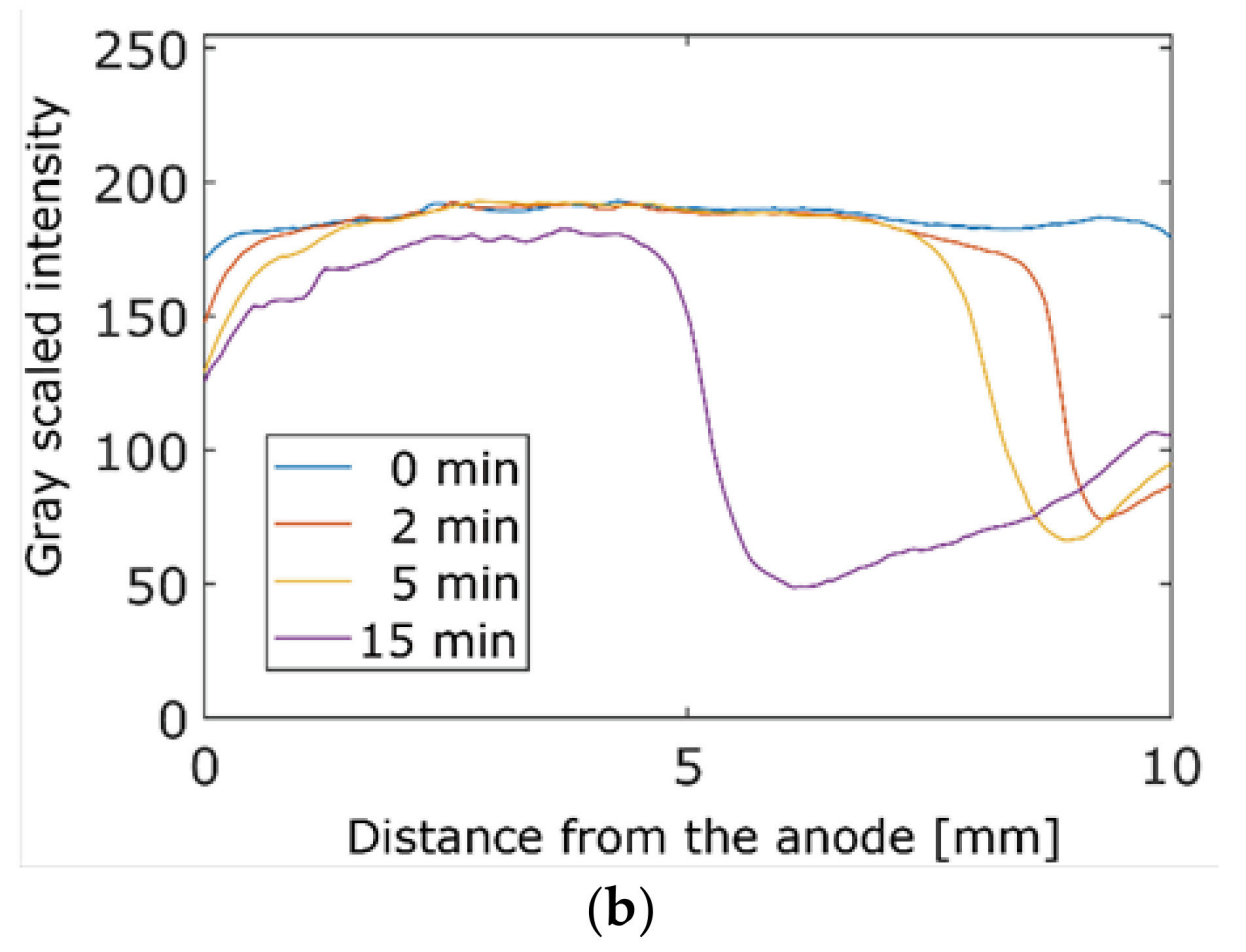
Time evolution of pH distribution after applying voltage to the buffer solution. (**a**) Typical photo images of the pH test paper. (**b**) Gray-scaled intensity distribution between the electrodes.

We first visualized the time evolution of the pH distribution using the pH test paper. The origami structure in Figure 1c was temporary replaced by the pH test paper without folding. The pH test paper was completely wetted with the buffer solution, and the voltage was applied. The result is shown in Figure 2a. We converted the RGB color image of Figure 2a to a gray-scaled image by the rgb2gray function implemented in MATLAB 2017. The gray-scaled intensity distributions between the electrodes are shown in Figure 2b. This figure approximately shows the color change due to the variation of pH. The intensity of about 190 corresponds to pH 7; the intensity decreases with varying the pH. The ions in the buffer solution migrated in response to the electric field; thus, the pH distribution varied with time in the paper. The pH near the center of the pH test paper was kept at ca. 7 when the run time was less than 5 min. When the run time was 15 min, the anode and the cathode sides of the paper were changed to pH 2 and pH 12, respectively, because of the electric field. Since the charge of ampholytes such as amino acids depends on the pH, this result indicated that it was difficult to separate ampholytes by electrophoresis with a long run time.

### 3.2. pI Marker Separation

As shown in Section 3.1, the separation of ampholytes should be carried out in a short run time (less than 5 min), while the pH distribution is preserved. We propose the above-mentioned origami structure. Since the distance between the electrodes was small (10 mm), a high electric field of (5.0 × 10^2^ V/m) was obtained even with a portable power bank of 5 V. Moreover, the separation distance could be extended by expanding the origami structure.

**Figure 3:**
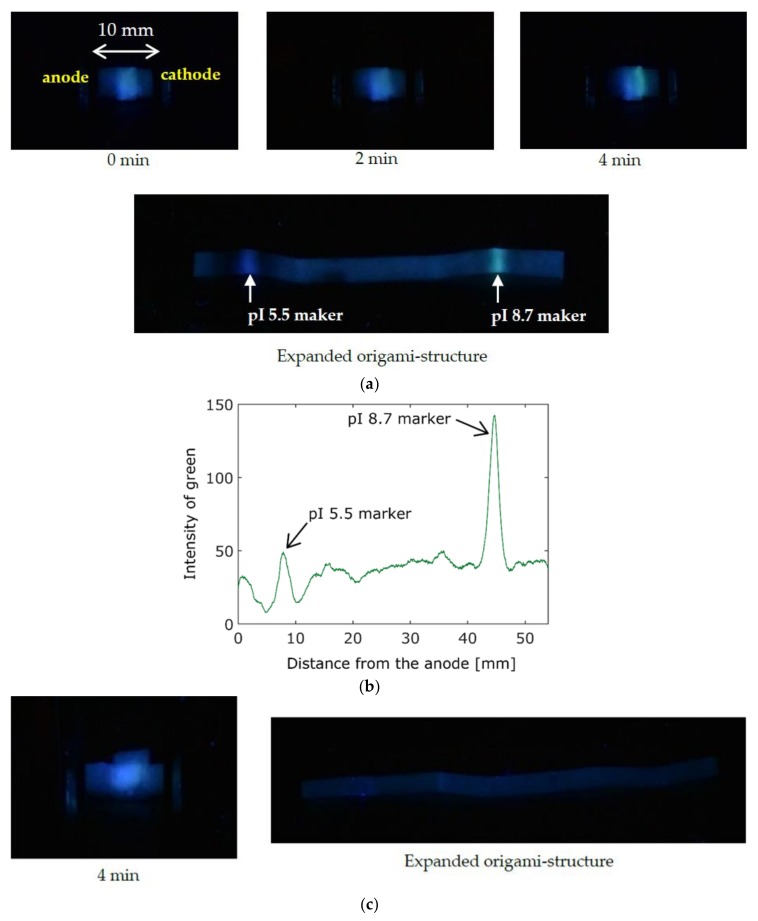
Fluorescent images of pI 5.5 and pI 8.7 markers during electrophoresis. (**a**) Time-lapse images during electrophoresis at 5 V. (**b**) Intensity distribution of the green color along the extended paper. (**c**) Control experiment in the absence of applied voltage (0 V).

We demonstrated the electrophoretic separation on the proposed origami structure. First, the origami structure was completely wetted with the buffer solution. Second, the sample solution (4 μL) was dropped on the sample pad, which was placed at the center of the origami structure, by using a pipette (Pipetman P2, Gilson, Middleton, WI, USA). Third, the portable power bank was used to apply a voltage (5 V) between the electrodes. The fluorescent images (blue fluorescence: pI 5.5, green fluorescence: pI 8.7) taken during the electrophoresis are shown in Figure 3. We used the sample solution consisting of pI 5.5 and 8.7 markers and the buffer solution at pH 7.0; thus, the pI markers moved in opposite directions. The long run time (>15 min) will lead the markers to the sample pad again because of the variation in time of the pH distribution, as shown in Section 3.1 (pH < 5.5 on the left side and pH > 8.7 on the right side, as shown in Figure 2).

As shown in Figure 3a, the pI 5.5 and pI 8.7 markers slightly moved toward the left (anode) and right (cathode), respectively. The migration of both markers stopped after 4 min, and we interrupted the power supply from the portable power bank. The pI markers were separated after 4 min; however, we observed that the separation distance was small, as shown in Figure 3a. By expanding the origami structure, the separation distance was extended to about 40 mm, as shown in Figure 3a. Using a buffer solution with a pH value intermediate between the pI values of the target materials, the materials having lower and higher pI values would migrate in opposite directions; thus, our PAD enabled us to separate materials with short run time and low applied voltage. Figure 3b shows the intensity distribution of the green color extracted from the RGB image along the expanded paper. The intensity of the blue color was not considered because the effect of the illumination light was strong. The emission from the pI 8.7 marker was recognized at about 45 mm. This indicated that most of the pI 8.7 marker moved to the cathode. Since the green sensor of the camera was sensitive to wavelengths from about 400 to 600 nm, the offset of the intensity was about 40, due to the effect of the illumination light.

On the other hand, the markers did not migrate in the absence of the applied voltage. The fluorescence was very weak, because the markers stayed on the sample pad, as shown in Figure 3c.

## 4. Conclusions

We propose a PAD for the electrophoretic separation of ampholytes such as amino acids. The electrophoretic separation of ampholytes on paper is difficult, because the pH distribution on paper varies as a consequence of the electrophoresis of ions in the buffer solution. In a preliminary study, the pH distribution was visualized using a pH test paper and varied during 15 min of electrophoresis. The pH values near the anode and the cathode changed from 7 to about 2 and 12, respectively. Then, the separation should be carried out in a short run time. By folding the filter paper, a low operation voltage was achieved, where the power was supplied by a 5 V 1.5 A portable power bank through the USB type A receptacle. We successfully demonstrated the electrophoretic separation of pI markers (pI 5.5 and 8.7 markers) by our paper-based analytical device. The separation was achieved within 4 min before the pH distribution on the paper varied. While the separation distance on the paper was initially small, it was extended by expanding the origami structure. This result indicates that our proposed PAD is useful for electrophoretic separation.
